# A classification and characterization of two-locus, pure, strict, epistatic models for simulation and detection

**DOI:** 10.1186/1756-0381-7-8

**Published:** 2014-06-09

**Authors:** Ryan J Urbanowicz, Ambrose LS Granizo-Mackenzie, Jeff Kiralis, Jason H Moore

**Affiliations:** 1 Department of Genetics, Dartmouth College, 1 Medical Center Dr., Lebanon, NH 05001, USA

**Keywords:** Epistasis, Models, Simulation, Genetics, GAMETES, Computational geometry, Convex hull

## Abstract

**Background:**

The statistical genetics phenomenon of epistasis is widely acknowledged to confound disease etiology. In order to evaluate strategies for detecting these complex multi-locus disease associations, simulation studies are required. The development of the GAMETES software for the generation of complex genetic models, has provided the means to randomly generate an architecturally diverse population of epistatic models that are both pure and strict, i.e. all *n* loci, but no fewer, are predictive of phenotype. Previous theoretical work characterizing complex genetic models has yet to examine pure, strict, epistasis which should be the most challenging to detect. This study addresses three goals: (1) Classify and characterize pure, strict, two-locus epistatic models, (2) Investigate the effect of model ‘architecture’ on detection difficulty, and (3) Explore how adjusting GAMETES constraints influences diversity in the generated models.

**Results:**

In this study we utilized a geometric approach to classify pure, strict, two-locus epistatic models by “shape”. In total, 33 unique shape symmetry classes were identified. Using a detection difficulty metric, we found that model shape was consistently a significant predictor of model detection difficulty. Additionally, after categorizing shape classes by the number of edges in their shape projections, we found that this edge number was also significantly predictive of detection difficulty. Analysis of constraints within GAMETES indicated that increasing model population size can expand model class coverage but does little to change the range of observed difficulty metric scores. A variable population prevalence significantly increased the range of observed difficulty metric scores and, for certain constraints, also improved model class coverage.

**Conclusions:**

These analyses further our theoretical understanding of epistatic relationships and uncover guidelines for the effective generation of complex models using GAMETES. Specifically, (1) we have characterized 33 shape classes by edge number, detection difficulty, and observed frequency (2) our results support the claim that model architecture directly influences detection difficulty, and (3) we found that GAMETES will generate a maximally diverse set of models with a variable population prevalence and a larger model population size. However, a model population size as small as 1,000 is likely to be sufficient.

## Background

The phenomenon of epistasis, or gene-gene interaction, confounds the statistical search for *main effects*, i.e. single locus associations with phenotype [1]. The term *epistasis* was coined to describe a genetic ‘masking’ effect viewed as a multi-locus extension of the dominance phenomenon, where a variant at one locus prevents the variant at another locus from manifesting its effect [2]. In the context of statistical genetics, epistasis is traditionally defined as a deviation from additivity in a mathematical model summarizing the relationship between multi-locus genotypes and phenotypic variation in a population [3]. Alternate definitions and further discussion of epistasis is given in [1,4-9].

Limited by time and technology, and drawn by the appeal of “low hanging fruit”, it has been typical for genetic studies to focus on single locus associations (i.e. main effects). Unfortunately, for those common diseases typically regarded as complex (i.e. involving more than a single loci in the determination of phenotype) this approach has yielded limited success [10,11]. The last decade has seen a gradual acknowledgment of disease complexity and greater focus on strategies for the detection of complex disease associations within clinical data [1,12-14]. Beyond the detection of complex multilocus genetic models, theoretical investigations have also pursued their enumeration, generation, and classification. These theoretical works seek to lay the foundation for the identification and interpretation of multilocus associations as they may appear in genetic studies.

A natural stepping stone towards understanding complex multilocus effects is the examination of two-locus models. Early on, Neuman and Rice [15] considered epistatic two-locus disease models for the explanation of complex illness inheritance, highlighting the importance of looking beyond a single locus. Li and Reich [16] classified all 512 fully penetrant two-locus models, in which genotype disease probabilities (i.e. penetrances) were restricted to zero and one. This work emphasized diversity of complex models beyond the typical two-locus models previously considered by linkage studies. Of these models, only a couple exhibit what was later referred to as “purely” epistatic interactions. *Pure* refers to epistasis between *n* loci that do not display any main effects [13,17-20]. Alternatively, *impure* epistasis implies that one or more of the interacting loci have a main effect contributing to disease status [19,20]. Hallgrimsdottir and Yuster [21] later expanded this two-locus characterization to include models with continuous penetrance values. Within a population of randomly generated two-locus models, they characterized 69 “shape-based” classes of impure epistatic models. In addition, they observed that the “shape” of a model (1) reveals information about the type of gene interaction present, and (2) impacts the power (i.e. frequency of success) in detecting the underlying epistasis.

Taking aim at pure epistasis, Culverhouse *et. al.*[18] described the generation of two to four-locus *purely* epistatic models and explored the limits of their detection. Working with a precisely defined class of models such as pure epistasis offered a more mathematically tractable set for generation and investigation. The value of their work was not to suggest that purely epistatic models necessarily reflect real genetic interactions, but rather the ability extrapolate their findings to more likely epistatic models possessing small main effects.

Similar to these earlier works, the present study focus on statistical epistasis, which is the phenomenon as it would be observed in case-control association studies, quantitative trait loci (QTL) mapping, or linkage analysis. Exclusively, we focus on a precise subclass of epistasis which we refer to as pure and strict. *Strict*, conceptually alluded to in [18], refers to epistasis where *n* loci are predictive of phenotype but no proper multi-locus subset of them are [19,20]. Of note, all two-locus purely epistatic models are strict by default since no other subsets are possible with only two-loci. The loci in pure, strict models could be viewed as “fully masked” in that no predictive information is gained until all *n* loci are considered in concert. Therefore these models may be considered “worst case” in terms of detection difficulty. While this exact, extreme class of models is unlikely to be pervasive within real biological associations, they offer a gold standard for evaluating and comparing strategies for the detection and modeling of multiple predictive loci.

A handful of studies have introduced methods for generating epistatic models [18,22-24] including our own Genetic Architecture Model Emulator for Testing and Evaluating Software (GAMETES) [19] designed to randomly generate an architecturally diverse population of pure, strict, epistatic models. *Architecture* references the unique composition of a model (e.g. the particular penetrance values and arrangement of those values across genotypes). Additionally, in [20] an Ease of Detection Measure (EDM) was introduced and incorporated into GAMETES offering a predictor of model detection difficulty calculated directly from the penetrance values and genotype frequencies of a given genetic model. Previously we demonstrated that a 2-locus model’s EDM was more strongly and significantly correlated with the detection power than heritability or any other metric considered. Detection power was determined separately using three very different, cutting edge data search algorithms in order to establish EDM calculation as a simple alternative to completing model detection power analyses.

In the present study we refine the characterization of two-locus models described in [16] and [21] to a more specific subset of models defined as having pure, strict, epistasis. We generate these models using GAMETES [19,20] and apply the geometric approached used in [21] to similarly identify shape model classes. Next, we examine whether model EDM scores (a surrogate measure of detection difficulty) differs between these shape groups as well as between groups with the same number of edges in their projected shapes. Then, we evaluate the impact of GAMETES model population as well as the effect of fixing population prevalence (K) or allowing it to vary randomly on observed model shape coverage and EDM score range. This study expands our theoretical understanding of a particularly challenging class of multi-locus models and suggests novel insight into the effective generation of complex models with GAMETES.

## Methods

In this section, we describe (1) the modeling of epistasis with GAMETES, (2) the triangulation of model shape (3) our experimental evaluation.

### Modeling 2-Locus pure strict epistasis

Single nucleotide polymorphisms or (SNPs) are loci in the DNA sequence which can serve as markers of phenotypic variation. The term *genotype* has been used to refer both to the allele states of a single SNP, as well as the combined allele states of multiple SNPs. Herein, we will refer to the latter as a multi-locus genotype (MLG) whenever necessary.

Penetrance functions represent one approach to modeling the relationship between genetic variation and a dichotomous trait. Penetrance is the probability of disease, given a particular genotype or MLG. Our models assume Hardy Weinberg equilibrium such that, the allele frequencies for a SNP may be used to calculate it’s genotype frequencies as follows; freq(*AA*) = *p*^2^, freq(*Aa*) = 2*pq*, and freq(*aa*) = *q*^2^, where *p* is the frequency of the major (more common) allele ‘*A*’, *q* is the minor allele frequency (MAF) where ‘*a*’ is the minor allele, and *p* + *q* = 1. Penetrance functions may be constructed to describe *n*-locus interactions between *n* predictive loci using a penetrance function comprised of 3^*n*^ penetrance values corresponding to each of the 3^*n*^ MLGs.

Table 1 gives an example of an epistatic model that is both pure and strict. For convenience all values in the table have been rounded to three decimals places. While fully penetrant models, like the ones characterized in [16] are easy to interpret, they are rarely representative of real world relationships between genotype and disease. A common example of a fully penetrant, purely epistatic 2-locus model based on the XOR function is given in Table 2. More realistic models, like the one in Table 1 and the ones typically generated by GAMETES, possess penetrance values between 0 and 1. Each of the nine entries in Table 1 corresponds to one of the nine possible MLGs combining SNPs 1 and 2. For instance, subjects that have the MLG *aa-bb* have a 14.7*%* chance of having disease. What makes these penetrance functions purely epistatic is that while the genotypes of SNPs 1 and 2 are together predictive of disease status, neither is individually. Further discussion of what makes models purely and strictly epistatic is given in [19].

**Table 1:** A 2-locus purely epistatic penetrance function

		**SNP 2**			**Marginal**
	**Genotype**	**BB (.25)**	**Bb (.5)**	**bb (.25)**	**Penetrance**
	AA (.36)	.266	.764	.664	.614
SNP 1	Aa (.48)	.928	.398	.733	.614
	aa (.16)	.456	.927	.147	.614
	Marginal	.614	.614	.614	K =.614
	Penetrance				

**Table 2:** Classic, fully penetrant 2-locus model of pure epistasis

		**SNP 2**			**Marginal**
	**Genotype**	**BB (.25)**	**Bb (.5)**	**bb (.25)**	**Penetrance**
	AA (.25)	0	1	0	.5
SNP 1	Aa (.5)	1	0	1	.5
	aa (.25)	0	1	0	.5
	Marginal	.5	.5	.5	K =.5
	Penetrance				

The GAMETES strategy for generating random, *n*-locus, pure, strict epistatic models is briefly reviewed here. Each *n*-locus model is generated deterministically, based on a set of pseudo random parameters, a randomly selected direction, and specified values of heritability, MAFs, and population prevalence (K). The GAMETES algorithm first (1) generates 2^*n*^ random parameters and a random unit vector in $R^{2^{n}}\;$, then (2) generates a random pre-penetrance function by seeding these parameters using the unit vector, and then (3) uses a scaling function to scale the entries of this random pre-penetrance function to generate a random penetrance function. To obtain a random penetrance function having a specified heritability, or heritability and K, it further (4) scales the entries of this penetrance function to achieve, if possible, these values. If steps (1) or (4) are not successful the algorithm starts over, attempting to generate models until either the desired model population size or the iteration limit is reached. For a detailed explanation of this strategy see [19].

EDM is utilized by GAMETES to select model architectures that span the range of predicted difficulties [20]. This allows for the design of a simulation study which diversifies model architecture based on detection difficulty. First we generated a population of pure, strict, epistatic models of random architecture sharing commonly specified genetic constraints (i.e. number of loci, heritability, MAFs, and K). GAMETES allows the user to specify a population size of models from which some will be selected to generate simulated genetic datasets. Certain constraint combinations may yield few or no viable models [19]. Therefore, GAMETES runs until either the desired population size or a maximum attempt limit is reached. Once one of the aforementioned stopping criteria is met, all models (each with the same constraints) were ordered by their EDM. At this point, GAMETES select some number of models to represent the range of observed EDMs. By default, GAMETES selects two models from this distribution, representing the highest and lowest EDM scores. A higher EDM indicates that a given model will be easier to detect than a model with a lower EDM. For the purposes of this study, we directed GAMETES to instead report the entire population of models generated by GAMETES.

### Shapes of two-locus models

The triangulation, or shape, of a model is used here to generalize it’s architecture and offer a classification of the type of interaction present. This geometric classification of epistasis was first applied to haploid models in [25], and extended to diploid two-locus QTL models in [21]. Overall, our approach was similar to [21], except that we used Qhull [26] as opposed to TOPCOM [27] to compute triangulations of the models.

**Figure 1:**
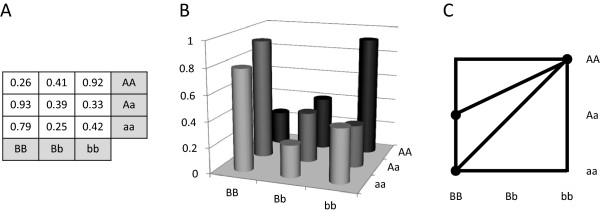
**Penetrance function projection.** Illustrating the projection of an arbitrary penetrance function model. **(A)** The penetrance function used. Note that this example model is not purely and strictly epistatic. **(B)** Bar plot of the penetrance values. Points would be placed in space at the top/center of each bar. **(C)** The projected 2-locus model shape given as a set of polygons.

Consider the example model given in Figure 1A. First, we place points in space where the *x* and *y* coordinates represent the 9 MLGs of this two-locus model and the *z* coordinates (or heights) of these points are the penetrance values at these MLG (see Figure 1B). Four additional points are placed at the outside corners of the x-y coordinates. Each additional point has an equal, negative height (not shown in Figure 1B). This was done so that Qhull could correctly discern the convex hull formed by these MLG heights. A model’s shape is defined by the upper faces of the convex hull of these heights. As explained in [21], this surface would intuitively be formed by draping a piece of stiff cloth over these points. The point coordinates are passed to Qhull [26] which determines the convex hull, and projects the upper faces (i.e, the creases of the surface) onto an *xy*-plane. This projection results in a set of polygons. Irrelevant polygons which include any of the four reference points of negative height are discarded. A unique set of polygons determines the classification of model shape (see Figure 1C). A mathematical definition of triangulation is given in [21].

As in [16] and [21] we take symmetry into account when defining shape classes. Symmetry is determined by (1) interchanging locus 1 and locus 2, or (2) interchanging two alleles at one or both loci. In [21], shape classes were further characterized by *circuits* (i.e. linear combinations of penetrance values) which decompose the main and epistatic effects of a model. In the present study, all models being classified are purely epistatic, having no main effects to decompose. Circuits could be used to decompose the types of interaction effects characterizing a model however this is beyond the scope of the present study.

### Experimental evaluation

We use GAMETES to generate differently sized populations of pure, strict, two-locus epistatic models possessing different constraint combinations. Specifically, populations were generated for heritabilities of 0.005, 0.01, 0.025, 0.05, 0.1, or 0.2, MAFs of 0.2 or 0.4 and with population prevalence (K) either fixed to 0.3 or allowed to vary to any value between 0 and 1. Thus, a total of 24 constraint combinations were considered (6 heritabilities, ∗ 2 MAFs ∗ 2 prevalence settings). Heritability and MAF constraints were seletect to be consistent with previous work using GAMETES [19,20], and the K value of 0.3 was selected based on the limits described in [19] to ensure that the specified combinations of heritability and MAF would yield models. We explore a variable K since a specific population prevalence rarely of interest in simulation studies, and previous findings in [19] indicated that a variable K facilitated viable model discovery in GAMETES.

For each constraint combination above, GAMETES was used to generate a population of models of sizes 1,000, 10,000, and 100,000 yielding a total of 72 different populations of models. All together, 2,664,000 models were generated which is similar in magnitude to the 1,000,000 random models examined in [21]. Within each of these 72 populations we characterize all model shapes as previously described. Additionally, we further generalize model shape by categorizing models by the number of edges as well as the number of polygons (triangles) existing within it’s shape class.

Observations in [21] suggested that the power to detect randomly generated, impure epistatic models was correlated with model shape. Extending these findings, we examine how model detection difficulty differs between shape classes observed in populations of pure, strict, epistatic models. We utilize EDM as a surrogate for detection difficulty or power, where power is used to describe the frequency of successful detection of a model. EDM is calculated directly from the penetrance function circumventing the need to generate simulated datasets and perform a secondary evaluation of power. The non-parametric Kruskal-Wallis test [28] was used to evaluate whether model EDM significantly differed within separate shape classes, as well as between groups defined by the number of edges in the model projection. Mann-Whitney pairwise comparisons were subsequently utilized to look for EDM differences between models with a specific number of projection edges.

## Results and discussion

**Figure 2:**
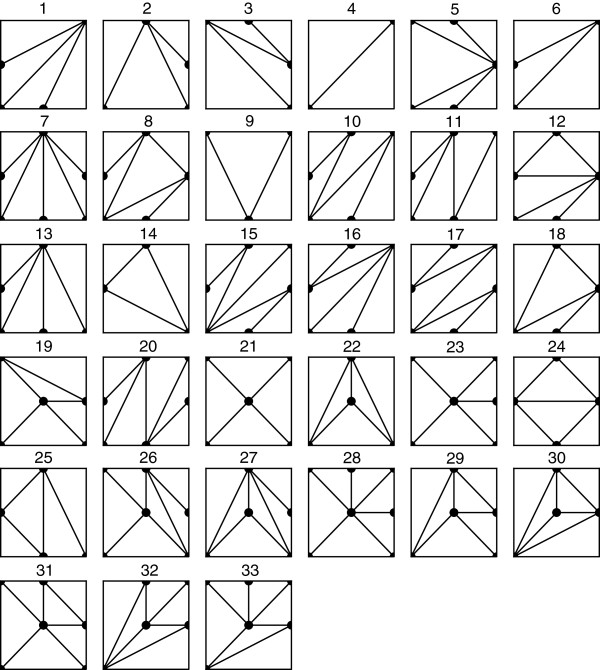
**Shape symmetry classes.** The 33 symmetry classes of the shapes of 2-locus pure strict epistatic models. The number above each model is it’s upper hull classification, uniquely identifying the shape throughout this study.

Across all 72 populations GAMETES-generated pure, strict, two-locus epistatic models, we identified 33 unique shape classes. This is in contrast to the 69 symmetry shape classes identified when not restricting models to pure, strict, epistasis [21]. It is important to note that pure, strict 2-locus epistastic models are not limited to these 33 shape classes, but rather that these are the only shape classes we observed when generating over two million genetic models with GAMETES. Case in point, we did not observe the shape class for the classic XOR penetrance function given in Table 2 (which would look like a baseball diamond including 4 edges). Strictly speaking, the XOR model diamond ‘shape’ is not a triangulation (since it is not comprised entirely of triangles), but rather it is a subdivision. Subdivisions, which are not triangulations, are unlikely to be generated randomly. Also note that shape class 24 in Figure 2 is a refinement of that subdivision. Models such as this, and potentially other unique shape classes, have an extremely low probability of being generated.

Figure 2 illustrates the projections which depict the 33 observed shape symmetry classes. The ID numbers assigned to shape symmetry classes were arbitrarily assigned according to the order in which the unique class was identified within the model populations. Notice that different shape triangulations possess different numbers of edges. For example, the only triangulation with a single edge is symmetry class 4, and the only triangulations with two edges are symmetry classes 6 and 9. We observe a maximum of 6 edges in our symmetry class projections. Shape classes are organized by number of edges in Table 3.

**Table 3:** Edge numbers in shape classes

**Number of edges**	**Associated class ID’s**
1	4
2	6,9
3	1,2,3,14
4	5,10,11,13,16,18,21,25
5	7,8,12,15,17,19,20,22,23,24
6	26,27,28,29,30,31,32,33

**Figure 3:**
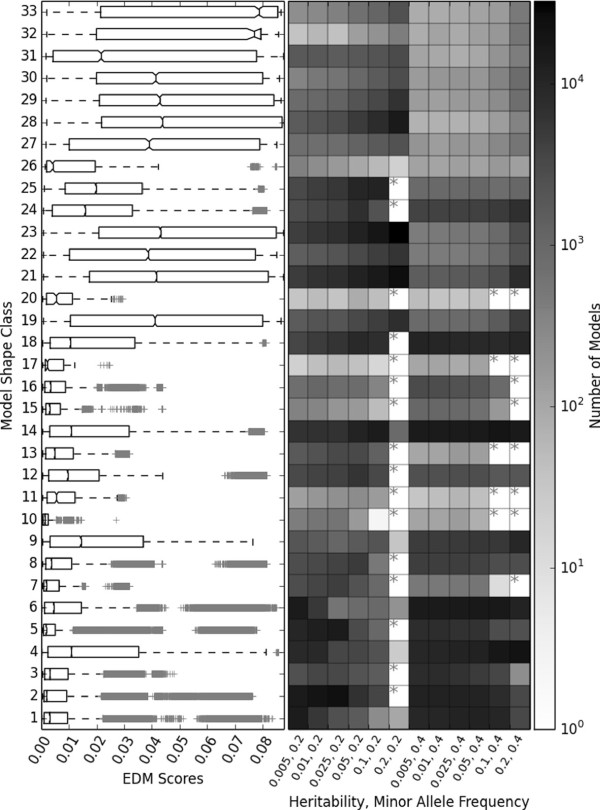
**Shape and EDM score distributions within 100,000 model populations.** A summary of shape classifications in 12 populations of 100,000 models with fixed K (0.3). The left side of the figure gives box plots summarizing the distribution of model EDMs observed in the 12 combined populations for each shape class. The model shape class IDs correspond to the symmetry classes given in Figure 2. The right side of the figure summarizes the number of models generated for each shape class in each of the 12 populations. The number of models is given on a logarithmic scale. Grey stars indicate that within the given model population, no models were found belonging to the respective shape class.

Towards the characterization of our now shape-classified models we first explore all 36 model populations with a fixed K. For each of the three model population sizes (1,000, 10,000 and 100,000) we examine the distribution of EDM scores obtained across all 12 constraint combinations of heritability and MAF. Similarly, we examine the number of models that have been randomly generated by GAMETES for each shape class. Figure 3 illustrates these findings for EDM distribution and frequency of shape class occurrence for population sizes of 100,000. Notice that the distribution of EDM scores as well as respective median values can be dramatically different from one shape class to another. Kruskal-Wallis testing confirmed that the EDMs of models found in different shape groups were significantly different (P <<0.001). This significance held for every population size examined and whether K was fixed or not. Figure 3 also illustrates the number of models identified within each of the 12 combination populations that belong to a respective shape symmetry class. Examining a column of boxes indicates the coverage of shape classes within one of the 12 populations. If a column has no *’s, than the respective model population included at least one model each of the 33 observed shape classes. If a row of boxes has no *’s than at least one model was found for the respective shape class no matter which of the 12 constraint combinations were used. This figure not only illustrates the variability of EDM between shape classes, but the relative likelihood that these shape classes will be randomly generated for different model constraint combinations. It may be interesting in follow up research to compare the frequency with which we observe specific real-world epistatic model shape classes relative to what has been observed through random generation. For reference, it may also be useful to note that in [20], we observed that for all models with an EDM greater than 0.01 the multifactor dimensionality reduction (MDR) software [29] had significant power (>80*%*) to detect them within a dataset including 20 attributes, and 800 samples.

**Figure 4:**
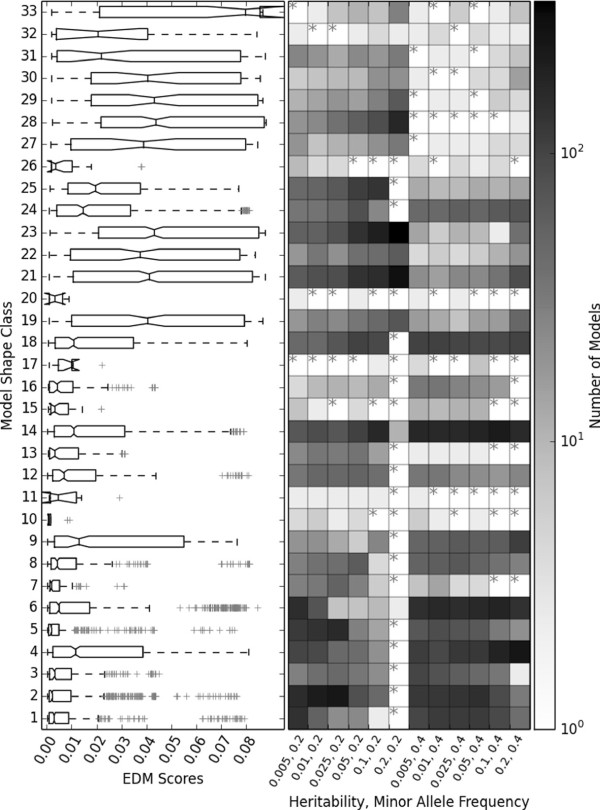
**Shape and EDM distributions within 1,000 model populations.** A summary of shape classifications in 12 populations of 1,000 models with fixed K (0.3). The left side of the figure gives box plots summarizing the distribution of model EDMs observed in the 12 combined populations for each shape class. The model shape class IDs correspond to the symmetry classes given in Figure 2. The right side of the figure summarizes the number of models generated for each shape class in each of the 12 populations. The number of models is given on a logarithmic scale. Grey stars indicate that within the given model population, no models were found belonging to the respective shape class.

Figure 4 offers an illustration identical to that found in Figure 3 except that only 1,000 models were generated for each of the 12 constraint combinations of heritability and MAF. The most obvious difference in comparing Figures 3 and 4, is that when a smaller model population size was used, the shape class coverage within each of the 12 populations decreased. In other words, as might be expected, the diversity of model shapes observed for different combinations of heritability and MAF became limited within a smaller population. Results for a population size of 10,000 fit this trend (See Figure S1 of the Additional file 1). Interestingly, Figure 4 also indicates that while some shapes were clearly less likely to be generated by GAMETES, all 33 shape classes were still represented within at least 1 of the 12 populations. Notably, in both the 1,000 and 100,000 population examples, specifying a heritability of 0.2 along with a MAF of 0.2 tended to particularly limit the diversity of model shapes that could be generated. Models belonging to shape classes 21 and 23 were instead dramatically more prevalent given these constraints.

Similar figures for populations of all three sizes and a variable K (instead of a fixed K) are given in the Additional file 1 (Figures S2, S3 and, S4). As for the fixed K populations, within the variable K populations Kruskal-Wallis testing confirmed that the EDMs of models found in different shape groups were significantly different (P <<0.001). However we observed one key difference when allowing K to vary. Specifically, the models generated were more evenly distributed across different shape classes, which simultaneously improved class coverage such that more shape classes were represented within each of the 12 constraint combination populations with variable K than when compared to fixed K. This finding is intuitive since allowing K to vary is less mathematically restrictive for penetrance function generation, and thus we would expect diversity.

**Figure 5:**
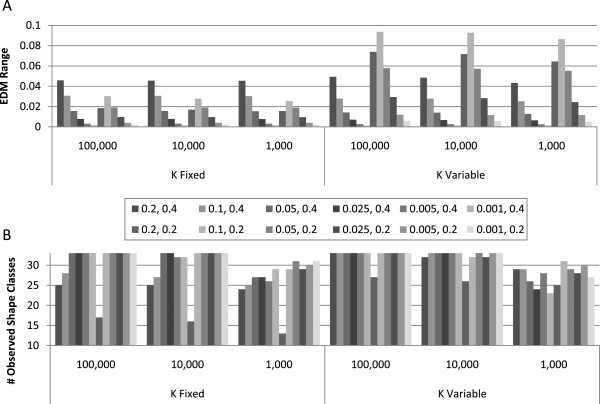
**EDM and shape classification coverage consistency.** Overall summary of EDM range and the number of shape classes observed within each of the 72 experimental populations. The legend differentiates each of the 12 combinations of heritability and MAF. **(A)** EDM range is the difference between the highest and lowest EDM value observed in models of the respective subpopulation. **(B)** This illustrates model coverage of the observed shaped classes for each population.

Figure 5 summarizes trends in both EDM scores and shape class coverage between all 72 populations. Specifically, Figure 5A summarizes the EDM range (i.e. the maximum EDM minus the minimum EDM observed within a respective population). Since GAMETES selects models representative of this model difficulty range, maximizing this range encourages the selection of best models to represent the easiest and most challenging models based on model architecture alone. This is valuable for developing the most thorough simulation study possible. Notice how for a fixed K, the EDM range largely consistent for different population sizes. However when comparing EDM ranges between fixed and variable K, we see that variable K can, for certain constraints (such as MAF = 0.2 and larger heritabilities), increase EDM range. Also, notice that EDM range tends to increase with heritability suggesting that architecture may be most important to consider for higher heritability models within simulation studies. Maximum and minimum EDM values for all populations are summarized in the Additional file 1: Figure S5. Figure 5B summarizes the shape class coverage for each of the 72 populations. This is the number of shape classes which were represented by models in the respective populations (where all 33 identified shape classes is the maximum). Observe that coverage tends to decrease along with population size whether K is fixed or not. However a variable K somewhat reduces loss in coverage.

**Figure 6:**
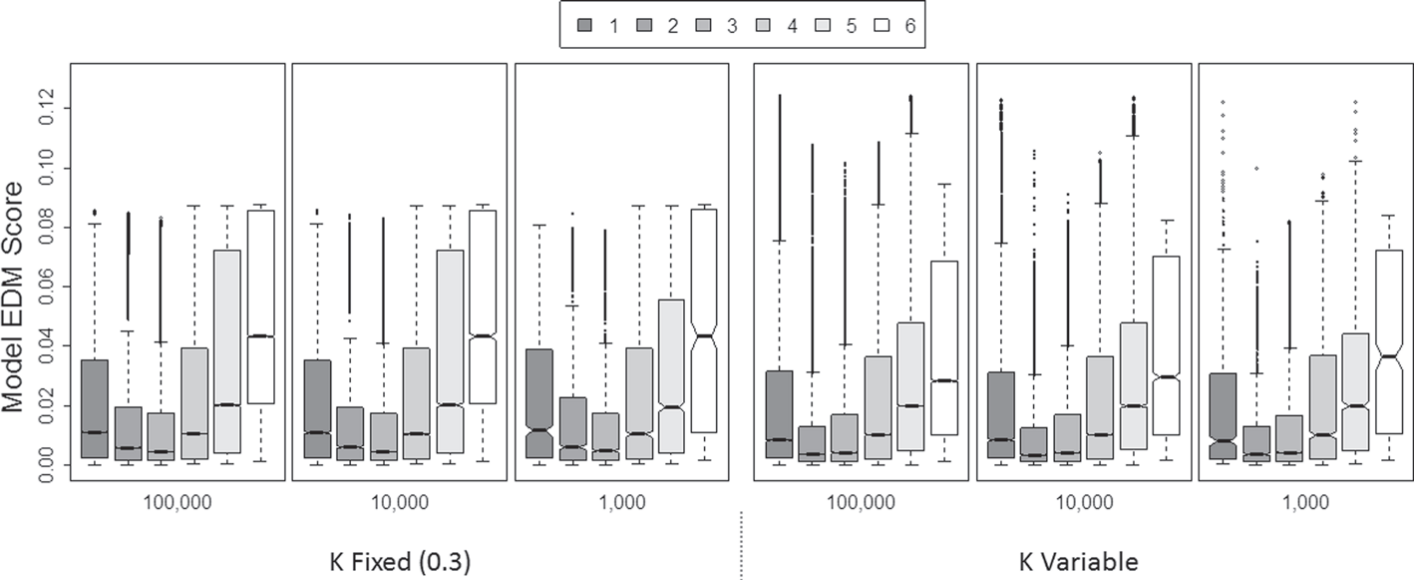
**Model detection difficulty (EDM) vs. number of edges in the model shape projection.** Box plots summarizing the distribution of EDM scores for models having anywhere from 1 to 6 edges in their respective shape projections. Each of the six boxes labeled by on of three population sizes (i.e. 100,000, 10,000, 1,000) incorporate all models from a respective set of 12 constraint combination populations, with either a fixed or variable K value.

Finally, we return to generalization of shape class projections by the number of edges or the number of triangles. While we considered both generalized classifications, here we only report the results for generalizing by edge number, as we found that it better captured underlying differences in detection difficulty (see Additional file 1 for the results of generalizing by number of triangles). Figure 6 explores trends in model EDM scores broken down by the number of edges in respective shape class projections. Keeping in mind that models with a higher EDM are generally easier to detect, models with 2 or 3 edges tend to be the most challenging to detect (lowest median EDM scores), models with 1 or 4 edges are somewhat easier to detect, and models with 5 than 6 edges are, on average, the easiest to detect. Kruskal-Wallis testing indicates that EDM scores are significantly different depending on the number of edges in the model (P <<0.001). Mann-Whitney pairwise comparisons reveal that nearly all pairwise comparisons between edge number groups as suggested by box-plot notches in Figure 6 (P <0.05) (see Additional file 1 for pairwise comparisons). The only exceptions are found for fixed K models in populations of 1,000 and 10,000 where the difference in average EDM between having 1 vs. 4 edges was not significant. Also of note, the proportion of models having a specific number of edges in their shape projections appears to be relatively stable, regardless of population size or how K is set (See Additional file 1: Figure S6). One other intuitive observation from this figure, is that when K is fixed at 0.3 the range of observed EDM scores is narrower than when GAMETES is allowed to explore a variable K. In [20] we had previously demonstrated while holding all other model constraints constant (i.e. heritability, minor allele frequency, and K), in inherent model ’shape’ could still significantly influence EDM. While beyond the scope of the present study, it might be interesting to further dissect the influence of model shape from that of K on a model’s detection difficulty.

## Conclusions

This study pursued three goals: (1) Classify and characterize pure, strict 2-locus epistatic models, (2) explore the relationship between model architecture and detection difficulty in the generalized context of model shape, and (3) explore the maintenance of model architecture diversity in GAMETES-generated model populations to establish guidelines for effective complex model generation. Our focus on such a precise, challenging class of epistatic models lends itself to both simulation studies, in which a gold standard for algorithmic evaluation is desirable, and to real world model detection where our characterization of a more mathematically tractable class of epistasis may facilitate the characterization of interaction in an observed biological model.

Our geometric classification of pure, strict, two-locus epistasis model shapes revealed 33 unique shape symmetry classes having 1 to 6 edges. This is in contrast to the 69 symmetry classes having 1 to 8 edges identified in randomly generated impure epistatic models [21]. The shape of a two-locus model may be used to classify and offer a visual representation of the type of gene interaction present. This classification of model shape might be applied to epistatic models identified in real world analyses in order to determine associated shape classes and EDM scores. It would be interesting to explore whether the model shapes of real world interactions tended to correspond with shapes that GAMETES generates more frequently by chance.

Having previously demonstrated the ability of EDM to predict detection power in [20], the present evaluation of model EDM transitively indicates that the shape of pure, strict epistatic models significantly influences detection difficulty. This is also in line with the claim made by [21] that an epistatic model’s shape impacts the power to detect it. Additionally, this finding highlights the importance of taking model architecture into consideration when generating models and datasets for the evaluation of algorithms. Further characterization of shape classes, grouped by number of edges in the shape projection, reveals that the number of edges alone is also predictive of relative detection difficulty.

The experimental variation of population size and K revealed the overall consistency of the GAMETES model generation strategy. GAMETES was designed to generate a random population of genetic models with diverse model architectures. We gauge this diversity on (1) the range of model EDMs (maximum and minimum observed EDM values), and (2) the number of shape classes observed in the generated population of models. Maintaining model diversity is ideal when constructing a simulation study which efficiently covers the space in which real biological disease associations could appear. Our results suggest that when generating models for a simulation study GAMETES effectively maintains diversity even at population size of only 1,000. However for maximal architectural diversity, a population size of 100,000 combined with a variable K is optimal. Combining GAMETES with geometric model classification could be used to select models for a simulation study which are both representative of each model class and representative of maximum and minimum EDM values. By developing better simulation study designs we hope to encourage the development of better complex model detection algorithms.

As suggested by [21], the geometric classification scheme applied in this paper could be expanded to three or more loci, as well as to QTL studies (by scaling penetrance values outside the range 0-1, such that values at MLGs become expected phenotype magnitudes). It is crucial not only to develop techniques for the detection of complex multilocus interactions, but to develop a theoretical understanding of epistasis. This work will promote effective simulation studies and facilitate the characterization of observed real-world interactions by better defining the charteristics of one group of complex epistatic models to which others may be compared.

## Abbreviations

SNP: Single nucleotide polymorphism; GAMETES: Genetic architecture model emulator for testing and evaluating software; MLG: Multi-locus genotype; MAF: Minor allele frequency; K: Population prevalence; EDM: Ease of detection measure.

## Competing interests

The authors declare that they have no competing interests.

## Author’s contributions

RU organized the analysis, carried out statistical analyses, and wrote the majority of the manuscript. DGM, coded the shape projection and classification strategy, carried out the experiments, developed key figures and co-wrote the manuscript. JK assisted with the shape projection strategy. JH co-wrote the manuscript. All authors read and approved the final manuscript.

## Supplementary Material

Additional file 1 **Supplemental materials.** Includes supplemental results and figures. Click here for file
