# Supplementary material for: A classification and characterization of two-locus, pure, strict, epistatic models for simulation and detection
Source: BioData Min. 2014 Jun 9;7:8. doi: 10.1186/1756-0381-7-8 (PMC4094921; doi:10.1186/1756-0381-7-8)
Supplement: Additional file 1 — Supplemental materials. Includes supplemental results and figures. [file 1756-0381-7-8-S1.pdf]

A Classification and Characterization of Two-Locus Pure, Strict Epistatic Models for Simulation and Detection - *Supplementary Materials*

Ryan J. Urbanowicz, Ambrose Granizo-Mackenzie, Jeff Kiralis, Jason H. Moore

March 18, 2014

In this document, we provide supplementary materials for the results section.

1 Results

Figure 1 summarizes model EDMs and frequency of shape occurrence for each shape class (for fixed K and population sizes of 10,000). This summary was quite similar to Figure 3 from the paper (which examines 100,000 model populations). However, notice that in Supplemental Figure 1, five fewer count cells have a '*', which indicates that over all 12 populations, 5 fewer shape classes were covered. Figures 2, 3, and 4 illustrate similar summaries for populations generated with a variable K, having population sizes of 100,000, 10,000, and 1,000, respectively. Figure 5 gives the maximum and minimum EDM values observed within each population. These values were used to specify the EDM Range summarized in Figure 5 of the paper.

As mentioned in the paper we considered both the number of edges and the number of triangles as a generalization of model shape to capture differences in model detection difficulty. Similar to Table 3 in the paper, Table 1 below gives the shape classes that belong to groups defined by a specific number of triangles in their respective shape projections. Note that the one shape with a single edge has two triangles, both shapes with two edges have three triangles, and all of the shapes with three edges have four triangles. Differently of the shapes with four edges, all have five triangles except shape 21, which has four triangles. Of the shapes with five edges, shapes 19, 22, and 23 have five triangles, while the rest have six. Lastly, all shapes with six edges also have six triangles. First, in Figure6 we report all pairwise Mann-Whitney statistical evaluations for shape generalization using edge number. Next in Figure 7 we report all pairwise Mann-Whitney statistical evaluations for shape generalization using the number of triangles in respective shape classes. Next, similar to Figure 6 in the paper, Figure 8 gives box plots summarizing the the distribution of EDM scores for models having anywhere from 2 to 6 triangles in their respective shape projections. You can see from this figure that by grouping models by the number of triangles in the shape class, we do not observe as dramatic a difference in EDM scores from group to group.

Figure 9 gives the ratio of models generated with a respective number of edges in it's respective shape projection for all populations generated. Figure 10 gives the same ratios, but for the number of triangles in respective shape projections.

Table 1: Triangle numbers in shape classes.

Number of Triangles	Associated Class ID's
2	4
3	6,9
4	1,2,3,14,21
5	5,10,11,13,16,18,19,22,23,25
6	7,8,12,15,17,20,24,26,27,28,29,30,31,32,33

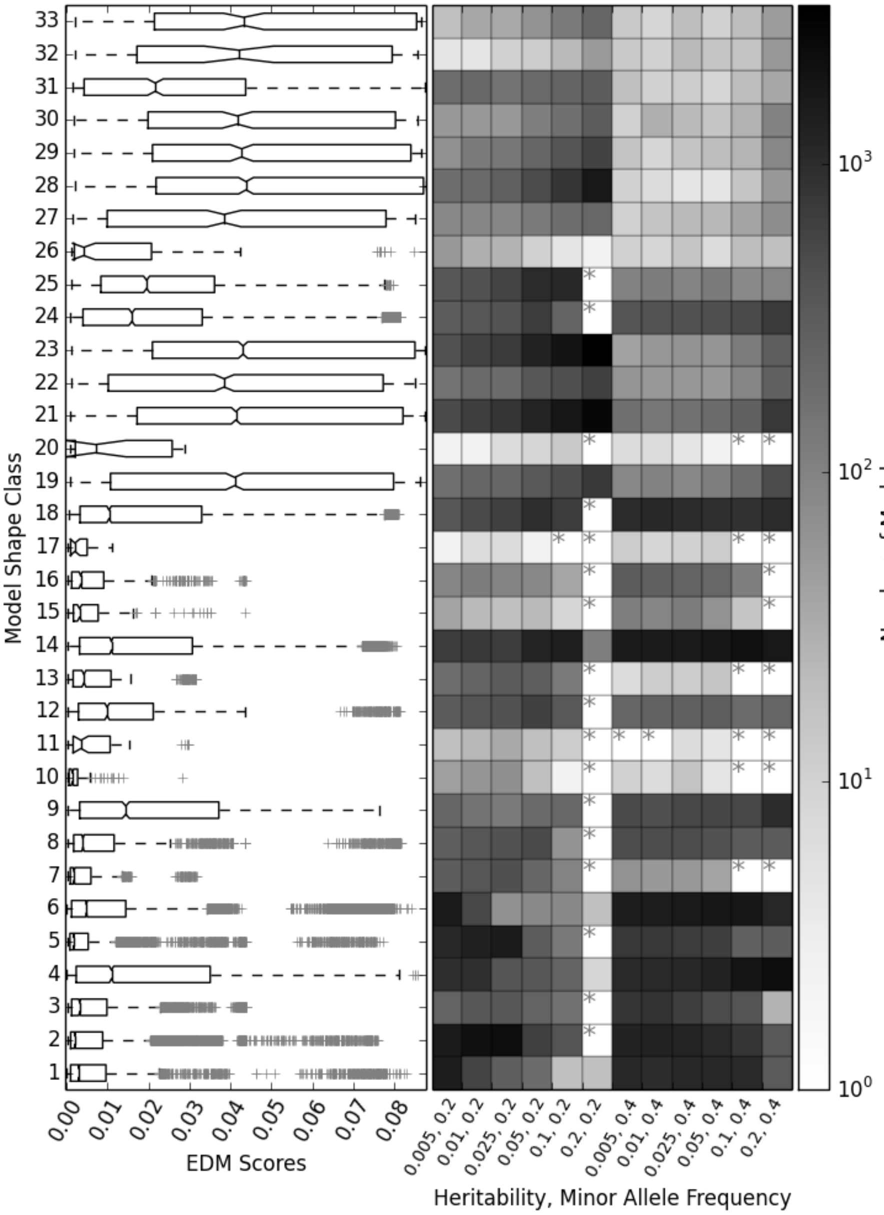

Figure 1: **Shape and EDM score distributions within 10,000 model populations.** A summary of shape classifications in 12 populations of 10,000 models with fixed K (0.3). The left side of the figure gives box plots summarizing the distribution of model EDMs observed in the 12 combined populations for each shape class. The model shape class IDs correspond to the symmetry classes given in Figure 2. The right side of the figure summarizes the number of models generated for each shape class in each of the 12 populations. The number of models is given on a logarithmic scale. Grey stars indicate that within the given model population, no models were found belonging to the respective shape class.

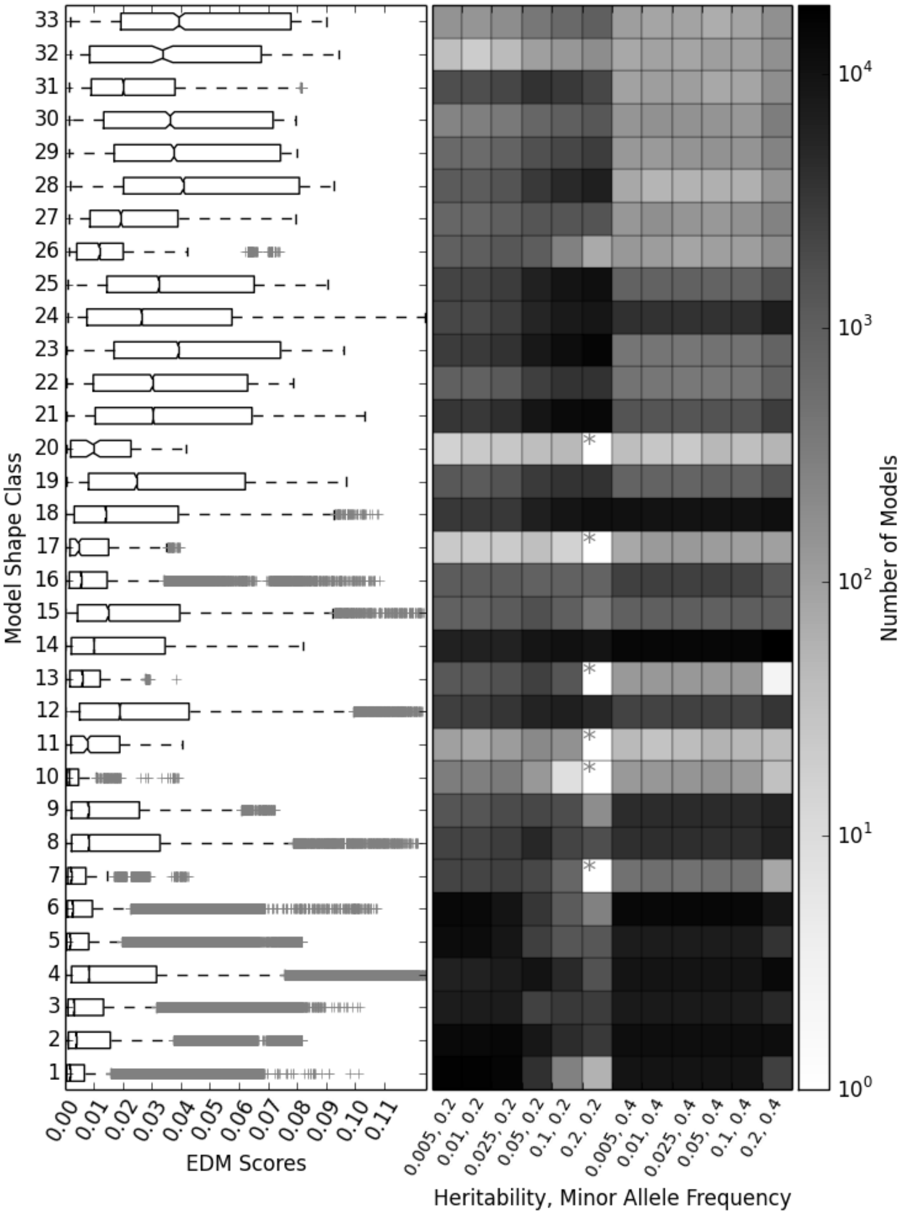

Figure 2: **Shape and EDM score distributions within 100,000 model populations.** A summary of shape classifications in 12 populations of 100,000 models with variable K. The left side of the figure gives box plots summarizing the distribution of model EDMs observed in the 12 combined populations for each shape class. The model shape class IDs correspond to the symmetry classes given in Figure 2. The right side of the figure summarizes the number of models generated for each shape class in each of the 12 populations. The number of models is given on a logarithmic scale. Grey stars indicate that within the given model population, no models were found belonging to the respective shape class.

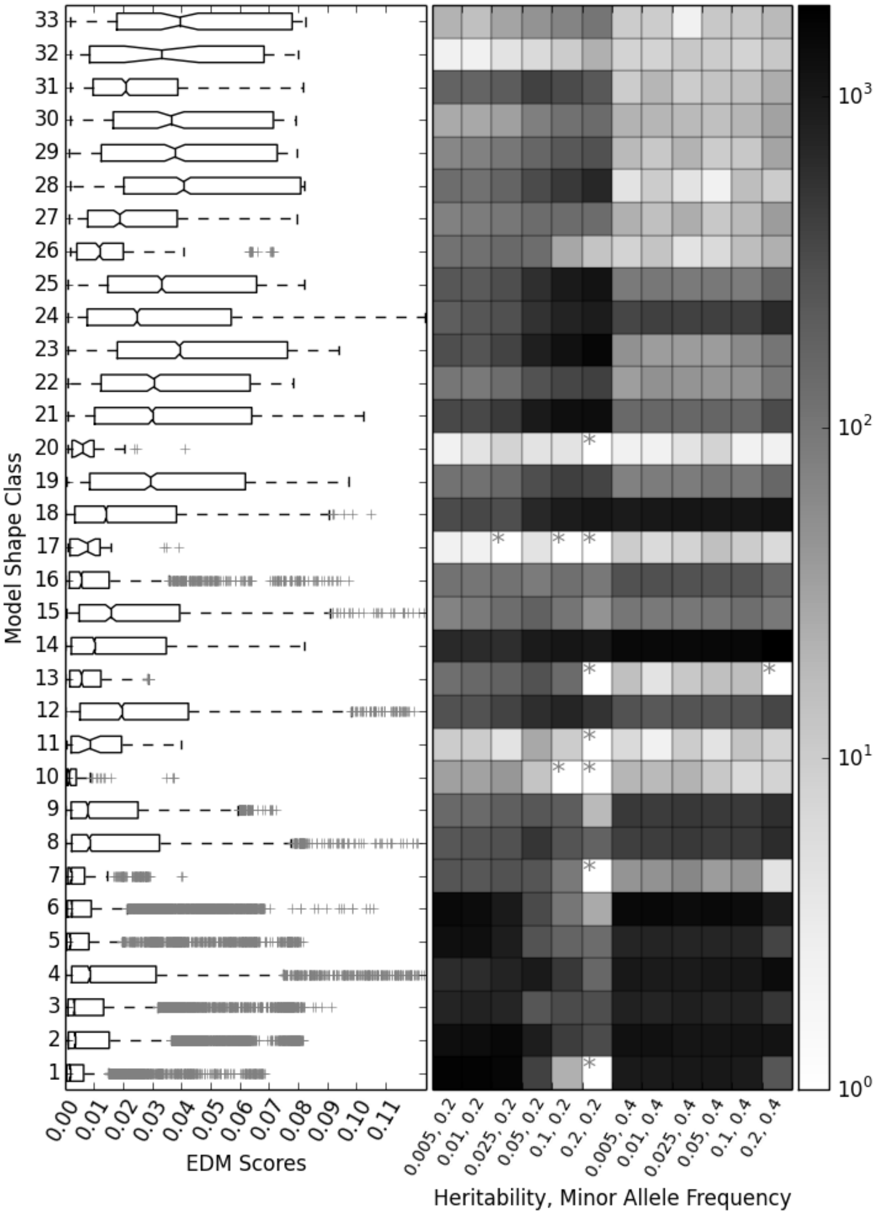

Figure 3: **Shape and EDM score distributions within 10,000 model populations.** A summary of shape classifications in 12 populations of 10,000 models with variable K. The left side of the figure gives box plots summarizing the distribution of model EDMs observed in the 12 combined populations for each shape class. The model shape class IDs correspond to the symmetry classes given in Figure 2. The right side of the figure summarizes the number of models generated for each shape class in each of the 12 populations. The number of models is given on a logarithmic scale. Grey stars indicate that within the given model population, no models were found belonging to the respective shape class.

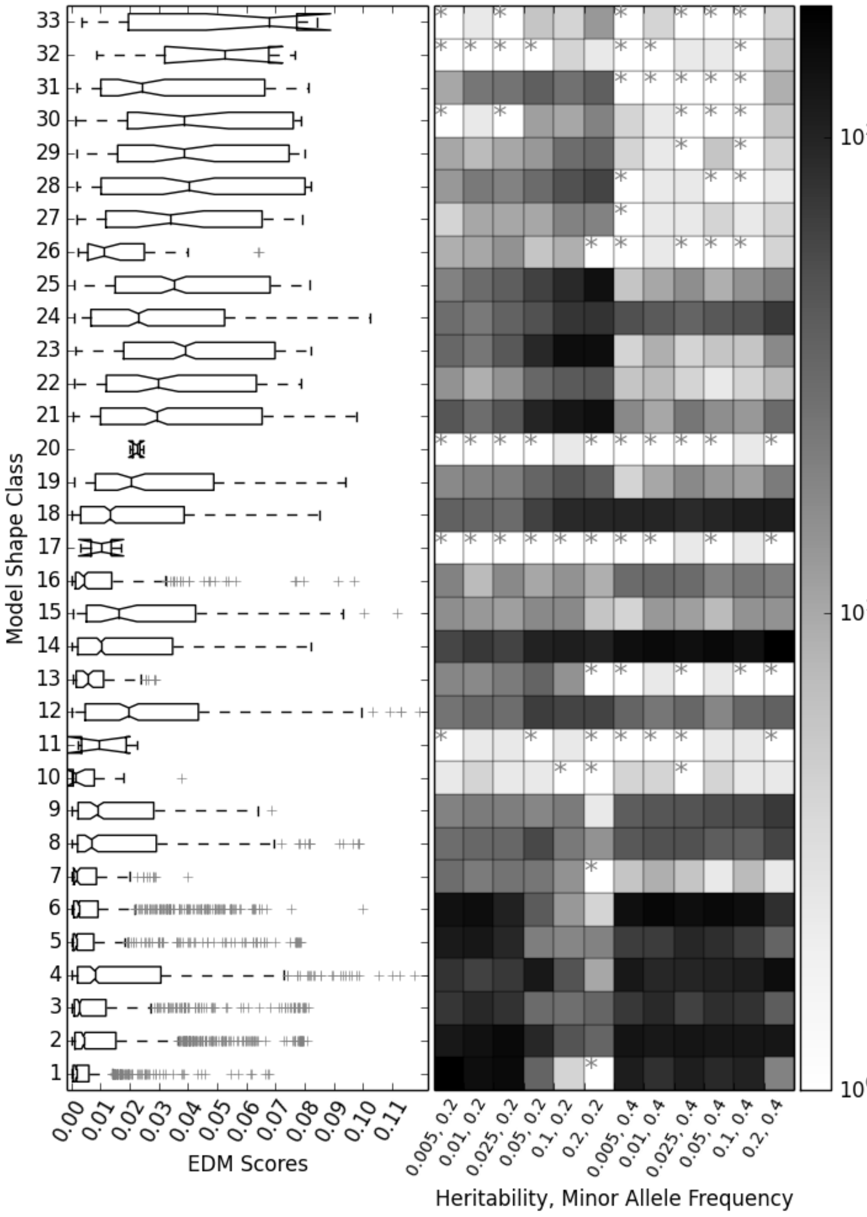

Figure 4: **Shape and EDM score distributions within 1,000 model populations.** A summary of shape classifications in 12 populations of 1,000 models with variable K. The left side of the figure gives box plots summarizing the distribution of model EDMs observed in the 12 combined populations for each shape class. The model shape class IDs correspond to the symmetry classes given in Figure 2. The right side of the figure summarizes the number of models generated for each shape class in each of the 12 populations. The number of models is given on a logarithmic scale. Grey stars indicate that within the given model population, no models were found belonging to the respective shape class.

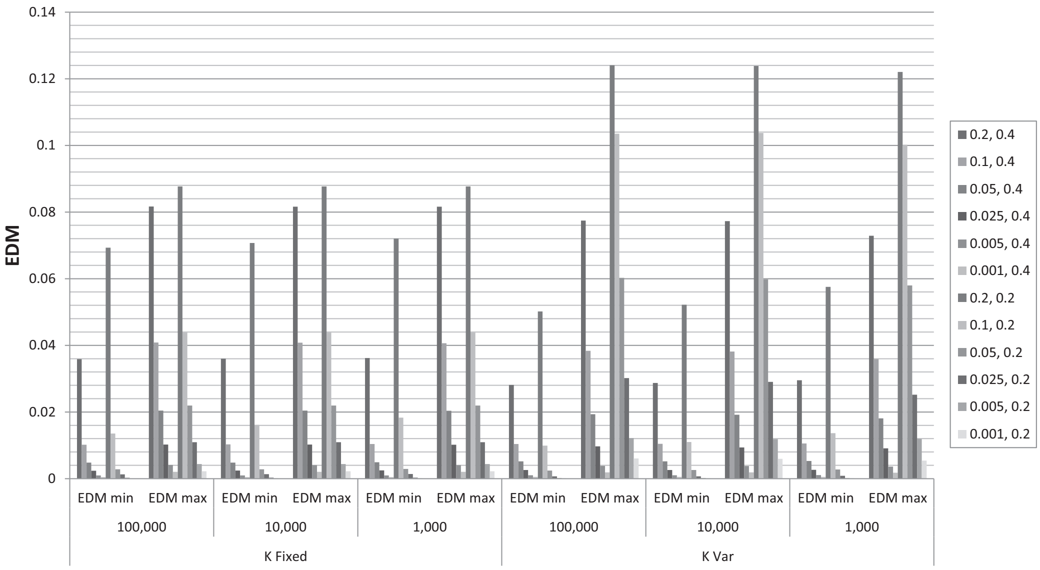

Figure 5: **Maximum and minimum EDM scores.** For all experimental populations, this figure gives the maximum and minimum EDMs observed. The legend differentiates each of the 12 combinations of heritability and mAF.

Mann-Whitney Pairwise Comparisons (Edges)

Prevalence (K)	Pop Size	Edges	1	2	3	4	5	6
0.3	100000	Edges	1	2	3	4	5	6
		1		2.20E-16	2.20E-16	5.01E-04	2.20E-16	2.20E-16
		2			2.20E-16	2.20E-16	2.20E-16	2.20E-16
		3				2.20E-16	2.20E-16	2.20E-16
		4					2.20E-16	2.20E-16
		5						2.20E-16
		6						
0.3	10000	Edges	1	2	3	4	5	6
		1		2.20E-16	2.20E-16	1.71E-01	2.20E-16	2.20E-16
		2			2.20E-16	2.20E-16	2.20E-16	2.20E-16
		3				2.20E-16	2.20E-16	2.20E-16
		4					2.20E-16	2.20E-16
		5						2.20E-16
		6						
0.3	1000	Edges	1	2	3	4	5	6
		1		3.44E-14	2.20E-16	6.13E-01	1.46E-14	2.20E-16
		2			5.26E-05	2.20E-16	2.20E-16	2.20E-16
		3				2.20E-16	2.20E-16	2.20E-16
		4					2.20E-16	2.20E-16
		5						2.20E-16
		6						
Variable	100000	Edges	1	2	3	4	5	6
		1		2.20E-16	2.20E-16	2.20E-16	2.20E-16	2.20E-16
		2			2.20E-16	2.20E-16	2.20E-16	2.20E-16
		3				2.20E-16	2.20E-16	2.20E-16
		4					2.20E-16	2.20E-16
		5						2.20E-16
		6						
Variable	10000	Edges	1	2	3	4	5	6
		1		2.20E-16	2.20E-16	2.20E-16	2.20E-16	2.20E-16
		2			2.20E-16	2.20E-16	2.20E-16	2.20E-16
		3				2.20E-16	2.20E-16	2.20E-16
		4					2.20E-16	2.20E-16
		5						2.20E-16
		6						
Variable	1000	Edges	1	2	3	4	5	6
		1		2.20E-16	2.20E-16	2.24E-02	2.20E-16	2.20E-16
		2			1.54E-04	2.20E-16	2.20E-16	2.20E-16
		3				2.20E-16	2.20E-16	2.20E-16
		4					2.20E-16	2.20E-16
		5						2.41E-13
		6						

Figure 6: **Mann-Whitney pairwise evaluations:Number of Edges.**

Mann-Whitney Pairwise Comparisons (Triangles)

Prevalence (K)	Pop Size	Triangles	2	3	4	5	6
0.3	100000	Triangles	2	3	4	5	6
		2		2.20E-16	2.20E-16	2.20E-16	2.20E-16
		3			2.20E-16	2.20E-16	2.20E-16
		4				2.20E-16	2.20E-16
		5					2.20E-16
		6					
0.3	10000	Triangles	2	3	4	5	6
		2		2.20E-16	2.20E-16	2.20E-16	2.20E-16
		3			2.20E-16	2.20E-16	2.20E-16
		4				2.20E-16	2.20E-16
		5					2.20E-16
		6					
0.3	1000	Triangles	2	3	4	5	6
		2		3.44E-14	2.27E-11	4.79E-03	2.28E-04
		3			5.06E-03	2.20E-16	2.20E-16
		4				2.20E-16	2.20E-16
		5					5.08E-02
		6					
Variable	100000	Triangles	2	3	4	5	6
		2		2.20E-16	2.20E-16	2.20E-16	2.20E-16
		3			2.20E-16	2.20E-16	2.20E-16
		4				2.20E-16	2.20E-16
		5					2.20E-16
		6					
Variable	10000	Triangles	2	3	4	5	6
		2		2.20E-16	2.20E-16	2.20E-16	2.20E-16
		3			2.20E-16	2.20E-16	2.20E-16
		4				2.20E-16	2.20E-16
		5					2.20E-16
		6					
Variable	1000	Triangles	2	3	4	5	6
		2		2.20E-16	1.33E-12	1.41E-05	2.20E-16
		3			9.62E-14	2.20E-16	2.20E-16
		4				2.20E-16	2.20E-16
		5					4.58E-11
		6					

Figure 7: **Mann-Whitney pairwise evaluations:Number of Triangles.**

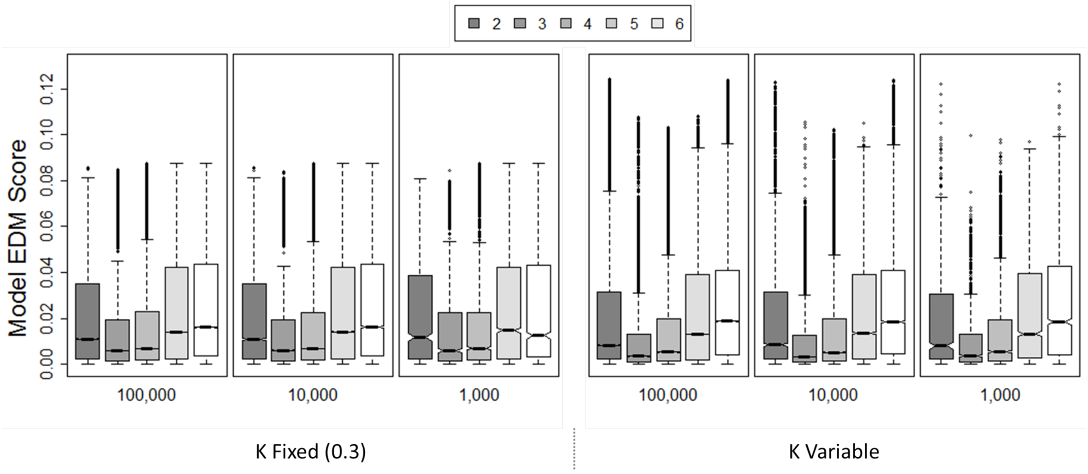

Figure 8: **Model detection difficulty (EDM) vs. number of triangles in the model shape projection.** Box plots summarizing the distribution of EDM scores for models having anywhere from 2 to 6 triangles in their respective shape projections. Each of the six boxes labeled by on of three population sizes (i.e. 100,000, 10,000, 1,000) incorporate all models from a respective set of 12 constraint combination populations, with either a fixed or variable K value.

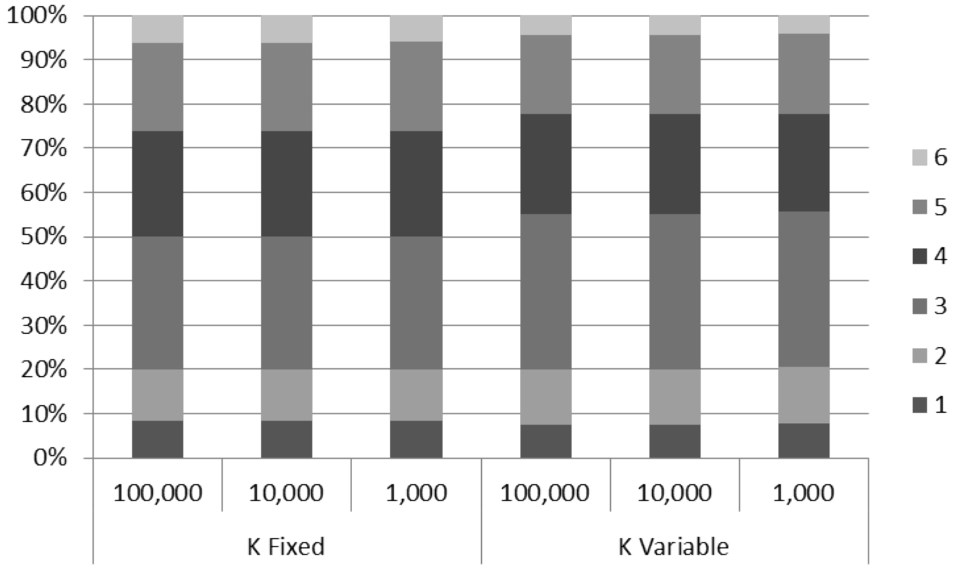

Figure 9: **Ratio of models generated with different numbers of edges in their projected shapes.** Each bar, divided by the grey-scale reflects the respective population size indicated below it. Each division illustrates the proportion of models that were generated which yielded a respective number of edges in the shape projection (anywhere from 1-6 edges were observed).

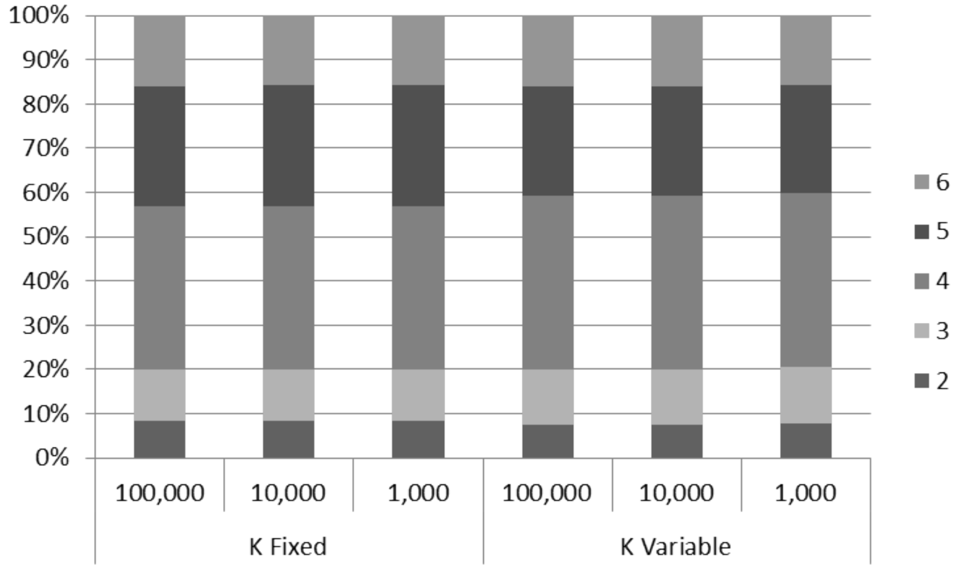

Figure 10: **Ratio of models generated with different numbers of triangles in their projected shapes.** Each bar, divided by the grey-scale reflects the respective population size indicated below it. Each division illustrates the proportion of models that were generated which yielded a respective number of triangles in the shape projection (anywhere from 2-6 triangles were observed).
